# Insight into the Formation of Glimepiride Nanocrystals by Wet Media Milling

**DOI:** 10.3390/pharmaceutics12010053

**Published:** 2020-01-09

**Authors:** Djordje Medarević, Svetlana Ibrić, Elisavet Vardaka, Miodrag Mitrić, Ioannis Nikolakakis, Kyriakos Kachrimanis

**Affiliations:** 1 Department of Pharmaceutical Technology and Cosmetology, Faculty of Pharmacy, University of Belgrade, Vojvode Stepe 450, 11221 Belgrade, Serbia; djordje.medarevic@pharmacy.bg.ac.rs (D.M.); svetlana.ibric@pharmacy.bg.ac.rs (S.I.); 2 Department of Pharmaceutical Technology, School of Pharmacy, Aristotle University of Thessaloniki, 54124 Thessaloniki, Greece; evardaka@pharm.auth.gr (E.V.); yannikos@pharm.auth.gr (I.N.); 3 Vinča Institute of Nuclear Sciences, University of Belgrade, Mike Petrovića Alasa 12–14, 11000 Belgrade, Serbia; mmitric@vinca.rs

**Keywords:** glimepiride, nanocrystals, wet media milling, energy vector diagrams, intermolecular interactions, crystal morphology

## Abstract

Nanocrystal formation for the dissolution enhancement of glimepiride was attempted by wet media milling. Different stabilizers were tested and the obtained nanosuspensions were solidified by spray drying in presence of mannitol, and characterized regarding their redispersibility by dynamic light scattering, physicochemical properties by differential scanning calorimetry (DSC), FT-IR spectroscopy, powder X-ray diffraction (PXRD), and scanning electron microcopy (SEM), as well as dissolution rate. Lattice energy frameworks combined with topology analysis were used in order to gain insight into the mechanisms of particle fracture. It was found that nanosuspensions with narrow size distribution can be obtained in presence of poloxamer 188, HPC-SL and Pharmacoat^®^ 603 stabilizers, with poloxamer giving poor redispersibility due to melting and sticking of nanocrystals during spray drying. DSC and FT-IR studies showed that glimepiride does not undergo polymorphic transformations during processing, and that the milling process induces changes in the hydrogen bonding patterns of glimepiride crystals. Lattice energy framework and topology analysis revealed the existence of a possible slip plane on the (101) surface, which was experimentally verified by PXRD analysis. Dissolution testing proved the superior performance of nanocrystals, and emphasized the important influence of the stabilizer on the dissolution rate of the nanocrystals.

## 1. Introduction

The oral route of administration is considered as the first choice for drug administration due to its convenience, non-invasiveness, good patient compliance and the lowest cost of therapy. However, absorption of drug after oral delivery can be significantly hindered if the drug is poorly soluble in the gastrointestinal fluids, since drug dissolution is necessary prerequisite for its absorption into systemic circulation. It is estimated that about 70% of newly synthesized drugs are poorly soluble in the aqueous fluids and this number steadily increases [1]. Increase in the number of poorly soluble drugs forces pharmaceutical industry to invest considerable efforts in the development of therapeutic systems for delivery of such drugs. Numerous approaches have been applied to overcome problems in oral delivery of poorly soluble drugs, such as salt formation [2], formulation of solid dispersions [3] and lipid based drug delivery systems [4], inclusion complexation with cyclodextrins [5,6], particle size reduction [7], etc. However, these techniques require some special features, (e.g., presence of acidic or basic groups, solubility in oils or organic solvents, suitable molecular size to fit into cyclodextrin cavity) [8] and usually require addition of high amount of excipients, making them unsuitable for high dose drugs. Nanosuspensions attracted considerable attention during the past two decades, as a useful cost-effective approach, applicable to almost all drugs. Nanosuspensions can significantly improve drug solubility and bioavailability, with high drug loading/administration at the same time. Additional benefits characteristic for nanosuspensions include increase of saturation solubility, as postulated by Ostwald-Freundlich equation and increase adhesiveness to biological membranes, which increase drug uptake via gastrointestinal tract [9,10]. Generally, nanosuspensions can be produced using two different approaches, based on completely inverse phenomena. Bottom up approach (i.e., precipitation) involves dissolving of drug in suitable solvent followed by controlled precipitation upon addition of anti-solvent and stabilizer. On the contrary, top down approach involves disruption of drug crystals under applied attrition force. Drawbacks of bottom up approach, such as, high amount of organic solvents and difficulties to control the particle size, as well as crystallinity and stability issues [11], shifted the focus of pharmaceutical industry to the development of top down techniques, which resulted in several products on the markets in the last years [12,13].

Among the drugs whose poor water solubility is a limiting factor to their bioavailability and therapeutic efficacy, and which are expected to benefit from a nanocrystal-based formulation, the antidiabetics of the sulfonylurea class hold a prominent position. Therefore, in the present study, glimepiride, a third-generation sulfonylurea oral hypoglycemic agent used for the treatment of patients with type II non-insulin-dependent diabetes mellitus [14], was selected as a model drug for nano-comminution. Since it exhibits very low solubility at acidic and neutral pH (<0.004 mg/mL) and high permeability through Caco-2 monolayer (30.4 × 10^6^ cm/s), glimepiride is classified in the class II of the Biopharmaceutics Classification System (BCS) [15], showing 100% absorption after oral administration [14,16]. However, its low water solubility and dissolution rate cause slow onset of action and variable bioavailability and unpredictable drug plasma levels following oral administration, which can lead to the absence of therapeutic response and expose the patient to serious hyperglycemia [17]. Numerous different approaches have been applied to improve the dissolution rate and bioavailability of glimepiride, including the use of co-solvents [18], self-emulsifying drug delivery systems [19], complexation with cyclodextrins or hydrotropic agents [17], micronization [20], solid dispersions with different hydrophilic polymers [20,21,22,23], and microencapsulation by spray congealing technology using hydrophilic meltable carriers [24].

Although there are several reports regarding the use of nanosuspensions for improving solubility and bioavailability of glimepiride [25,26], in these studies a precipitation method was used for nanosuspension preparation, which is not particularly suitable for large scale production. The suitability of hydroxypropyl cellulose, polyvinylpyrrolidone and poloxamers for nano-comminution of glimepiride by low energy wet media milling has been previously demonstrated [27]. However, this study did not deal with mechanisms responsible for crystal breakage during milling and capability of these systems to enhance dissolution rate of glimepiride. Additionally, to our knowledge, no study so far has provided any insight to the fracture mechanism of glimepiride crystals on the basis of intermolecular interactions in the crystal lattice. Therefore, the present study investigates the use of nanosuspensions stabilized by different hydrophilic polymers as an approach to improve the dissolution rate of glimepiride. Molecular modelling techniques were used in order to gain insight into the mechanisms that induce fracture of glimepiride crystals and are responsible for nanosuspension formation. High energy planetary ball milling was used for the production of nanosuspension, as this type of mill provides very high centrifugal forces to the milling beads, resulting in very high pulverization energy and short milling times.

## 2. Materials and Methods

### 2.1. Materials

**Figure 1:**
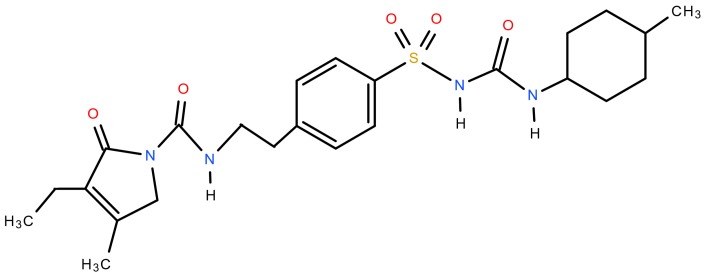
Chemical structure of glimepiride.

Glimepiride (Actavis, Leskovac, Serbia, chemical structure shown in Figure 1), was used as a poorly soluble drug. Two grades of hydroxypropyl cellulose-HPC-SL and HPC-L (Nisso HPC, Nippon Soda Co., Tokyo, Japan) and hypromellose (HPMC)-Pharmacoat^®^ 603 and Pharmacoat^®^ 615 (Shin-Etsu Chemical Co., Ltd., Tokyo, Japan), differing in viscosity, poloxamer 188 (Kolliphor™ P 188 micro, BASF, Ludwigshafen, Germany), polyvinylpyrrolidone K25 (PVP K25-Kollidon^®^ 25, BASF, Ludwigshafen, Germany) and polyvinyl caprolactam-polyvinyl acetate-polyethylene glycol graft copolymer (Soluplus^®^, BASF, Ludwigshafen, Germany) were tested as potential nanosuspension stabilizers. Mannitol (Pearlitol^®^ 160 C, Roquette Frères, Lestrem, France) was used as a matrix former for spray drying of the nanosuspension in order to prevent aggregation of glimepiride nanocrystals during solidification process.

### 2.2. Methods

#### 2.2.1. Preparation of Glimepiride Nanosuspensions

Glimepiride nanocrystal suspensions were prepared employing the wet media milling technique on a planetary ball mill (Pulverisette 7 Premium line, Fritsch GmbH, Idar-Oberstein, Germany). Glimepiride (0.5 g) and one of the tested stabilizers (25% *w*/*w* relative to glimepiride amount) were placed in 45 mL milling bowl loaded with 70 g of zirconium oxide milling beads (0.1 mm diameter). After addition 6 mL of water, milling was performed at 450 rpm mill rotation speed in 20 cycles of 3 min with 5 min breaks after each milling cycle to prevent instrumentation and sample overheating.

#### 2.2.2. Particle Size Measurements

The zeta average (z-average) size and polydispersity index (PDI) of glimepiride nanoparticles were monitored during the milling process by dynamic light scattering using a Zetasizer nano ZS instrument (Malvern Instruments, Malvern, UK). At predetermined time intervals (3, 6, 9, 15, 30, and 60 min), samples were withdrawn from the milling bowl, for the monitoring of particle comminution kinetics as a function of time. Each measurement was repeated in triplicate. Particle size determination was performed after a week’s storage in a refrigerator (5 ± 3 °C), in order to evaluate the short term stability of the nanoparticles.

#### 2.2.3. Spray Drying of Nanosuspensions

After selection of appropriate stabilizers, nanosuspensions were diluted with aqueous solution of mannitol (glimepiride:mannitol mass ratio 1:5) and spray dried using a Büchi B-191 Mini Spray-dryer (Büchi, Flawil, Switzerland) with the following process parameters: air flow rate 800 m^3^/h, inlet air temperature 80 °C (70 °C for formulation with poloxamer 188), aspirator 100% and pump speed of 5%. Spray dried samples were stored in a desiccator over phosphorus pentoxide until further analysis. Additionally, for comparison purposes, physical mixtures of glimepiride with mannitol and each stabilizer at the same mass ratio used in the milling experiments were prepared by manual mixing for 5 min, with mortar and pestle.

#### 2.2.4. Characterization of the Solidified Nanosuspensions

##### Redispersibility Testing

Redispersibility testing was performed in order to investigate the ability of reconstitution of glimepiride nanosuspension upon contact of solidified material with water. Approximately 2–3 mg of dried nanosuspension were immersed in 4 mL of distilled water and subjected to ultra-sonication for 3 min. The z-average diameter of the redispersed nanosuspensions was determined on a Zetasizer nano ZS particle size analyzer (Malvern Instruments, Malvern, UK), as previously described, and the Redispersibility Index (RDI) was calculated according to Equation (1) [28]:

(1) RDI (%) = (*D*_0_/*D*)

where *D*_0_ is the initial z-average diameter of the nanocrystals (before solidification), and *D* is the z-average diameter of the redispersed nanosuspension. RDI values close to 1 indicate that the nanosuspension can recover its original particle size after immersion to the aqueous medium.

##### Differential Scanning Calorimetry (DSC)

Accurately weighted 5–10 mg of samples were placed in perforated aluminium pans, and DSC scans were performed in the range of 25–250 °C at a heating rate of 10 °C/min, using a DSC 204 F1 Phoenix heat-flux differential scanning calorimeter (NETZSCH, Selb, Germany). Nitrogen gas flow (70 mL/min) was applied, and an empty pan was used as reference.

##### Attenuated Total Reflectance Fourier Transform Infrared (ATR-FTIR) Spectroscopy

FT-IR spectroscopy was used in order to detect the presence of intermolecular interactions between components of nanosuspensions. FT-IR spectra in the range of 600–4000 cm^−1^ at 4 cm^−1^ resolution were recorded using a horizontal Golden-Gate MKII single-reflection ATR accessory (Specac, Kent, UK) equipped with ZnSe lenses, mounted on a Shimadzu IR-Prestige-21 FT-IR spectrometer (Shimadzu Corporation, Kyoto, Japan). A total number of 32 scans was averaged per spectrum.

##### Powder X-ray Diffraction Analysis (PXRD)

Powder X-ray diffractometry (PXRD) scans were performed within the 3–40° 2*θ* range in 0.05° steps at a scan rate of 12 s per step, employing a Bruker D8 Advance diffractometer (Bruker, Karlsruhe, Germany) equipped with a Johanson type Ge-crystal primary monochromator producing CuK_α1_ radiation (*λ* = 1. 541 Å).

##### Scanning Electron Microscopy (SEM)

Nanocrystal samples were coated with a carbon later and SEM photomicrographs were acquired on a JSM 840A scanning electron microscope (JEOL, Tokyo, Japan).

##### In Vitro Dissolution Testing

Dissolution testing of glimepiride from the samples of pure drug and spray dried nanosuspensions was performed using rotating paddle apparatus during 3 h in 500 mL of phosphate buffer pH = 7.8, as recommended by FDA, with paddle rotation speed of 50 rpm. Sample mass equivalent to 6 mg of glimepiride was used for testing. At predetermined time intervals (5, 10, 15, 20, 30, 45, 60, 90, 120, 150, and 180 min) aliquots of 4 mL were withdrawn from the dissolution vessel, filtered through 0.1 μm membrane filter and the amount of dissolved glimepiride was determined spectrophotometrically at 226 nm. All analyses were performed in triplicate and results are expressed as mean ± standard deviation (SD).

#### 2.2.5. Computational Study of Glimepiride’s Crystal Properties

In order to enhance our understanding of glimepiride’s mechanical properties relevant to particle fracture during milling, the crystal properties of the commercially available polymorph I were modelled by a combination of quantum and molecular mechanics methods. The crystal structure of glimepiride polymorph I was retrieved from the Cambridge Structural Database (CSD reference code TOHBUN01) and all X–H bond lengths were normalized according to neutron diffraction data (C–H 1.083 Å and N–H 1.009 Å). Subsequently, various lattice properties and crystal morphology was calculated as follows:

##### Lattice Energy Frameworks

The total energy of the interactions of the basic molecule with its environment in the crystal was determined following the procedure described in the reference [29]. The first coordination sphere of glimepiride in the asymmetric unit, comprising molecules with atom-atom distance shorter than the van der Waals radii sum plus 1 Å for at least one pair of atoms, was determined, and the interaction energy for each dimer was calculated as the difference between the energy of a dimer and the energy of the constituting monomers. Density Functional Theory calculations were performed using the BLYP functional augmented by empirical dispersion correction (DFT-D) and def2-TZVP basis set, applying basis set superposition error correction with the Boys-Bernardi counterpoise procedure. The Orca quantum chemistry code [30] was used for the calculations, and the energy frameworks were constructed in the form of energy vector diagrams (EVDs) or “hedgehogs” representing the topology of intermolecular interactions in the crystals, using the CMOL collection of Python scripts for Energy Vector Diagram analysis of crystal structures [31]. The energy vectors originate from each molecule’s center of mass, and their length is directly proportional to the magnitude of the interaction energy between two molecules. The Mercury software program (Version 4.3.0) [32] was used for the visual representation of the EVDs.

##### Crystal Morphology

In order to understand how the lattice energy framework affects the crystals’ mechanical properties at the macroscopic level, the crystal morphology of glimepiride form I based on the surface attachment energy (SAE) theory, was calculated using the Oscail/Ritnos software program (Version 4.2) [33], using Lifson and Hagler potential parameters [34] in combination with Qeq atomic point charges [35] calculated using the GULP program (Version 3.0) [36].

## 3. Results

### 3.1. Wet Media Milling

**Figure 2:**
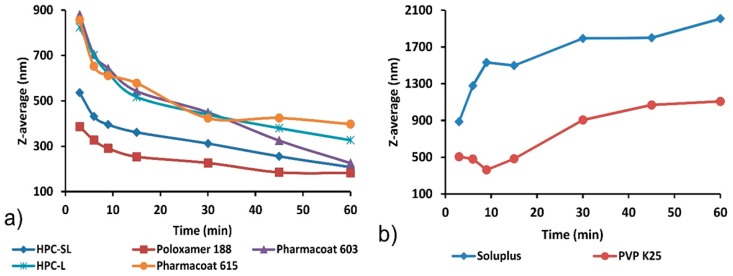
Glimepiride particle size vs. time profiles recorded during wet media milling with different stabilizers. (**a**) HPC-SL, HPC-L, Poloxamer 188, Pharmacoat^®^ 603 and Pharmacoat^®^ 615; (**b**) Soluplus^®^ and PVP K25. Polymers have been grouped according to Z-average diameter scale, for clarity.

Monitoring of particle size during milling process (Figure 2) confirmed generation of nanosuspension when poloxamer 188, HPC-SL, HPC-L, Pharmacoat^®^ 603 and Pharmacoat^®^ 615 were used as stabilizing agents. Measured particle size after 60 min of milling for these mixtures was in the range between 182.3 ± 11.8 and 397.7 ± 8.3 nm, while PDI for these experimental runs was below 0.3 (Table 1), indicating good particle size uniformity of the obtained nanosuspension [37].

These results proved that wet media milling using poloxamer 188, HPC-SL, HPC-L, Pharmacoat^®^ 603 and Pharmacoat^®^ 615 as stabilizing agents is a suitable technique for production of glimepiride nanosuspensions with uniform particle size. The milling process was most effective when HPC-SL and poloxamer 188 were used as stabilizing agents. When comparing particle size vs. time profiles of different grades of HPC and Pharmacoat^®^, it is obvious milling process is more effective with lower viscosity grades (HPC-SL and Pharmacoat^®^ 603). This is a consequence of viscous dampening effect where energy is dissipated by the displacement of the highly viscous phase, reducing the amount of energy which is transferred from the milling beads to the suspended particles [38]. This reduces the rate of fracture generation within the particles and decreases the kinetics of the milling process. The effect of viscosity on kinetics of milling process is particularly pronounced for planetary ball mills, since this type of mill does not contain external stirring device and the entire energy is provided to the milling beads by the centrifugal force, generated by rotating of the milling chamber [39]. Therefore, lower viscosity grades, HPC-SL^®^ and Pharmacoat^®^ 603, were selected for further studies.

Wet media milling process with PVP K25 resulted in initial particle size reduction during the first three cycles of milling (9 min), after which particle size increased until the end of the milling process (Figure 2b). When Soluplus^®^ was used as a stabilizer, the size of glimepiride crystals remained high and increased during the whole milling process, indicating particle aggregation and/or recrystallization. Particle growth in nanosuspensions can occur due to Ostwald ripening phenomenon where larger particles grow at the expense of dissolution of fine particles, which are more unstable in the suspending media due to their higher surface energy [40]. This surface energy difference provides the driving force for the migration of molecules from fines to larger particles. Supersaturation around large particles induces crystallization of drug and further particle growth [41]. Ostwald ripening can be considered as a mechanism of particle growth during milling of glimepiride, when PVP K25 and Soluplus^®^ were used as stabilizers, so these two polymers were excluded from further studies.

Since the prepared nanosuspensions were intended for further solidification after milling process, long term stability of liquid nanosuspensions was not required and therefore not tested. However, short-term stability after storage in the refrigerator was tested to show sensitivity of nanosuspensions to short storage periods between milling and solidification. Stability testing proved that initial particle size and polydispersity index remain almost unchanged after 7-day storage of nanosuspensions prepared with HPC, poloxamer 188, and Pharmacoat^®^ in the refrigerator, so the aforementioned nanosuspensions can be considered as sufficiently stable for industrial processing. The particle size reduction observed during storage of samples with Soluplus^®^ could be a result of further particle growth and consequent precipitation of excessively large particles, which can no longer remain dispersed and sampled for redispersibility testing. Based on the results of wet media milling experiments, nanosuspensions stabilized with HPC-SL (sample F1), poloxamer 188 (F2) and Pharmacoat^®^ 603 (F3) were selected for spray drying and further characterization.

### 3.2. Redispersibility Testing

Since liquid nanosuspensions are associated with physical stability issues, such as sedimentation, crystal growth (i.e., Ostwald ripening), aggregation and solid state transformation, solidification techniques are employed in order to develop stable dosage forms, suitable for commercial application [42]. Solidified nanosuspensions additionally provide an elegant dosage form with considerably reduced volume compared to liquid nanosuspensions and without special storage temperature requirements. However, the solidification process can often cause undesirable agglomeration of nanoparticles where reconstitution of the initial nanoparticles upon contact with an aqueous medium becomes impossible. In order to overcome this issue and ensure efficient nanoparticle recovery, water-soluble dispersants such as sugars (lactose, trehalose, sucrose), sugar alcohols (mannitol, xylitol), or cyclodextrins are commonly added to nanosuspensions before drying [43]. In the present study mannitol was used as a dispersant material. Redispersibility testing showed that the solidified nanosuspensions stabilized with HPC SL (RDI = 1.11) and Pharmacoat^®^ 603 (RDI = 1.04) releases initial nanoparticles after contact with aqueous media. Although the use of poloxamer 188 as a stabilizer resulted in the lowest size of glimepiride crystals after wet media milling process, the solidified sample of this nanosuspension failed to recover the initial nanoparticles (RDI = 0.53). The measured size of glimepiride nanoparticles after redispersion of this sample was around twice as large compared to nanosuspension before solidification. Since poloxamer 188 melts at low temperatures (~50 °C), agglomeration of particles occurs during spray drying in the stream of hot air, which was later proved by SEM analysis (Section 3.3.5).

### 3.3. Characterization of the Solidified Nanosuspensions

#### 3.3.1. Attenuated Total Reflectance Fourier Transform Infrared (ATR-FTIR) Spectroscopy

**Figure 3:**
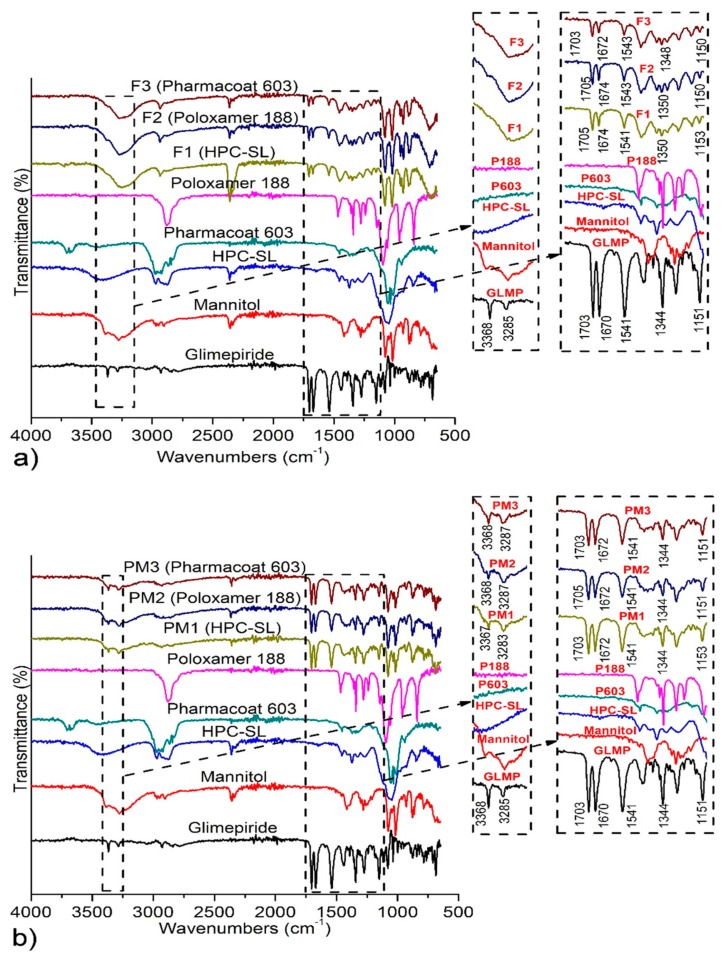
FT-IR spectra of: (**a**) Raw materials, spray dried nanosuspensions (F1–F3) and (**b**) corresponding physical mixtures (PM1–PM3). GLMP stands for Glimepiride, P603 for Pharmacoat^®^ 603, and P188 for poloxamer 188.

Figure 3 illustrates FT-IR spectra of raw materials and samples of solidified nanosuspensions and corresponding physical mixtures. Pure glimepiride exhibits characteristic absorption bands positioned at 3368 and 3285 cm^−1^ (ureidic N-H stretching vibrations), 1703 cm^−1^ (stretching vibrations of ureidic C=O group), 1670 cm^−1^ (stretching vibrations of amide C=O group), 1541 cm^−1^ (amide N–H bending vibrations), 1344 and 1151 cm^−1^ (vibrations of sulfonamide functional group), which is in accordance with the spectra previously described in the literature for polymorph I of glimepiride [22,44,45]. Spectra of physical mixtures correspond to the superposition of those of the individual components, indicating absence of chemical interactions between mixed components. The main difference in the position of characteristic glimepiride peaks between spectra of spray dried nanosuspensions and physical mixtures occurred in the region characteristic for ureidic N–H stretching vibrations. While these absorption bands are positioned at 3368 and 3285–3286 cm^−1^ in the spectra of pure glimepiride and physical mixtures, only a single broad band positioned between 3280 and 3180 cm^−1^ was observed in the spectra of spray dried nanosuspensions.

The observed changes of the shape and intensity of these absorption bands indicates some changes in the intermolecular interactions between glimepiride molecules, which can be induced by mechanical stress in the milling process. Significantly lower intensity in the IR absorption bands of glimepiride in the spectra of spray dried nanosuspensions, compared to those of corresponding physical mixtures gives further evidence of changes in the pattern of intermolecular interactions between glimepiride molecules in spray dried nanosuspensions. Glimepiride contains three hydrogen bond donors and seven hydrogen bond acceptors and it has been previously shown that glimepiride forms three intramolecular hydrogen bonds [46], while its crystal lattice contains strongly hydrogen-bonded dimers (structure of polymorph I, with CSD ref code TOHBUN01). Particle fracture resulting from collisions between milling beads and glimepiride crystals could alter the inter- and intra-molecular hydrogen bonding patterns, especially on the surface of glimepiride crystals, followed by stabilizer adsorption on the newly formed surfaces, could be the cause of the manifested changes in the FT-IR spectra.

#### 3.3.2. Differential Scanning Calorimetry (DSC)

**Figure 4:**
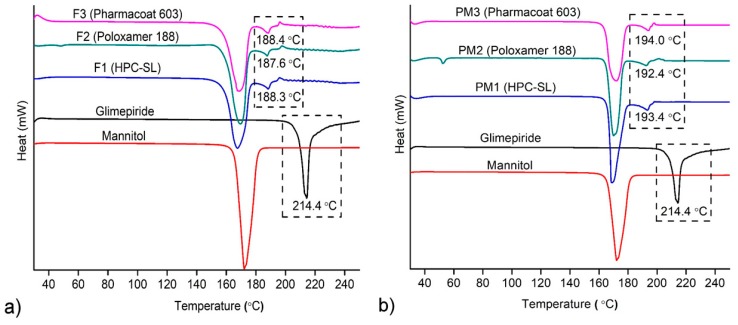
Differential scanning calorimetry (DSC) thermograms of: raw materials (**a**) spray dried nanosuspensions (F1–F3) and (**b**) corresponding physical mixtures (PM1–PM3).

DSC thermograms of raw materials, spray dried nanosuspensions and corresponding physical mixtures are shown in Figure 4. Pure glimepiride exhibits endothermic peak at 214.4 °C, corresponding to the melting of polymorph I [45].

All polymers used as stabilizers of nanosuspensions showed only broad endothermic events in the DSC curves, which are characteristic for amorphous materials (data not shown). The melting peak of glimepiride was observed in all samples of the spray dried nanosuspensions, confirming the presence of crystalline glimepiride. A significant shift of glimepiride’s melting peak from 214.4 °C to 187.6–188.4 °C was observed in the thermograms of spray dried nanosuspensions. It is well known that melting point depression occurs with reduction in the size of crystals, as described by the Gibbs–Thomson equation [47]. Additionally, mannitol as the major component of the mixture melts at lower temperatures and mixing of glimepiride with the molten mannitol also contributes to melting point depression phenomena. DSC analysis of physical mixtures of equivalent composition with the spray dried nanosuspensions also showed shifting of melting peak of glimepiride. However, in the case of spray dried nanosuspensions, glimepiride’s melting peak reduction was 5–6 °C lower, suggesting that this effect is a consequence of both crystal size reduction and mixing of glimepiride with molten mannitol.

#### 3.3.3. Powder X-ray Diffraction Analysis (PXRD)

**Figure 5:**
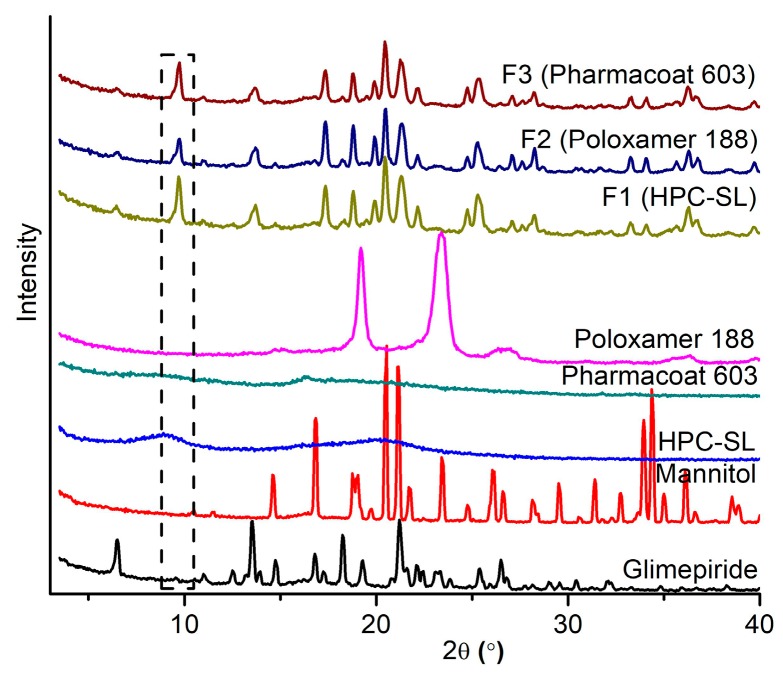
Powder X-ray diffraction (PXRD) patterns of raw materials and spray dried nanosuspensions.

PXRD analysis, Figure 5, was performed to test whether the wet media milling process induces polymorphic changes of glimepiride. The most pronounced peaks on the PXRD pattern of raw glimepiride were observed at 6.5, 13.55, 16.8, 18.25, and 21.2° 2*θ*, which correspond to those previously reported for the stable polymorph I of glimepiride [45,48].

All characteristic glimepiride peaks retained their positions in the diffractograms of spray dried nanosuspensions, indicating absence of polymorphic transitions during milling and spray drying. However, the peak positioned at 9.75° 2*θ*, which is very low in the diffractogram of pure glimepiride, significantly increased in intensity and became clearly pronounced in the diffractograms of all spray dried nanosuspensions. Changes of surface properties and crystal morphology due to the milling process have been previously documented for propranolol hydrochloride and paracetamol [49,50]. It is expected that milling process induces changes in crystal morphology, leading to overexpression of some crystal planes relative to the starting crystal morphology [51]. The changes in crystal morphology depend on the mechanisms of crystal fracture upon collision with the milling media. The highest probability for fracture generation in the crystal lattice is along the preferred slip plane. The 9.75° 2*θ* reflection corresponds to the (101) Miller plane. In order to elucidate the mechanism causing the increased expression of this particular Miller plane, the energy framework of the crystal lattice of glimepiride polymorph I, along with the crystal morphology model is discussed below.

#### 3.3.4. Lattice Properties and Morphology Calculations

**Figure 6:**
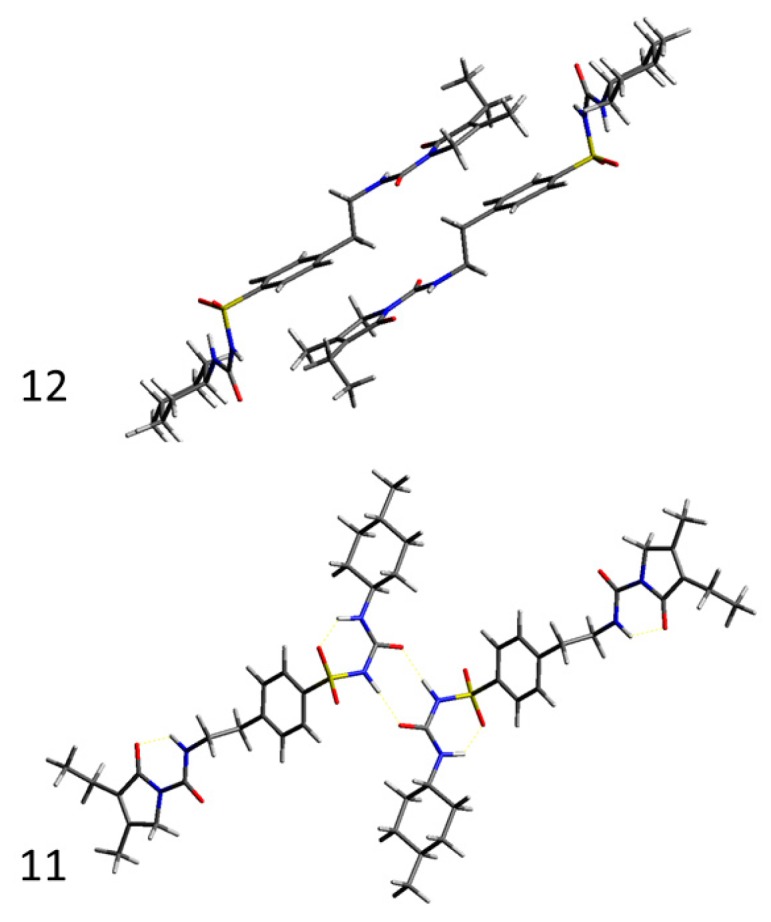
Illustration of the two strongest interacting dimers (11 and 12) in the crystal lattice of glimepiride.

**Figure 7:**
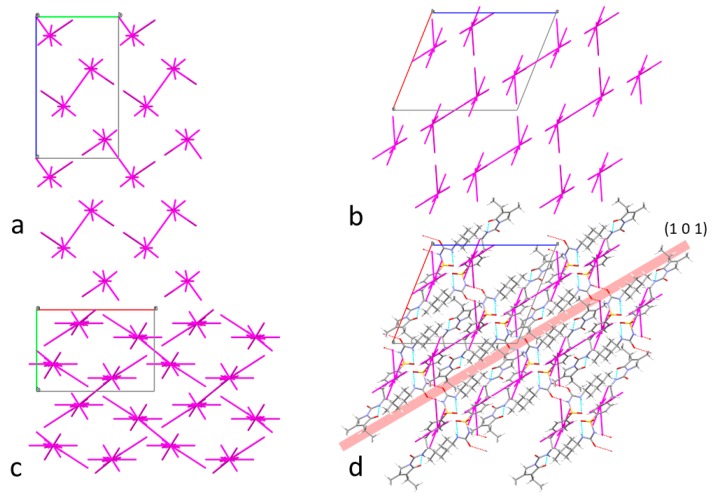
Energy vector diagrams (EVDs) plots viewed along the a—(**a**), b—(**b**), and c—(**c**) crystallographic axis together with an illustration of the (101) slip plane (**d**).

In an attempt to bridge the gap between macroscopic mechanical properties and crystal structure and lattice energetics, energy frameworks combined with topology analysis [52] were used as a means of slip plane identification in glimepiride crystals. Table 2 lists the intermolecular interaction energy of dimers formed by the basic molecule in the crystals, and the strongest interacting dimers are illustrated in Figure 6, while Figure 7 illustrates energy vector diagrams (EVDs or “hedgehogs”) of the intermolecular interactions in glimepiride’s crystal lattice viewed along the three crystallographic axes.

It is seen that the strongest stabilizing interactions (continuous connecting line between molecules in Figure 7) are formed between dimers interacting via their phenyl and pyrrole rings (dimer 12, *E*_inter_ = −21.5 kcal/mol), followed by hydrogen bonded dimers (dimer 11, *E*_inter_ = −17.5 kcal/mol). Inspection of the EVD plots alone reveals the existence of a slip direction along the (101) crystal plane, leading to the misconception that this could be a preferred slip plane that could facilitate plastic flow. A more detailed observation of the crystal lattice topology clearly shows that the (101) plane intersects with the intermolecular hydrogen bonds of dimer 11, therefore slip along that plane would require a substantial amount of energy in order to disrupt the hydrogen bonded dimers of glimepiride, rendering deformation by plastic flow rather unlikely, in favor of brittle fracture. Therefore, considering the spatial distribution of intermolecular forces depicted in the EVD plots, combined with topology analysis, it is expected that brittle fracture along the (101) crystal plane should occur upon the application of mechanical stress. This is in agreement with the PXRD findings that the (101) reflection overexpressed after milling, and it can be explained on the basis of preferred breakage of the particles along that plane.

**Figure 8:**
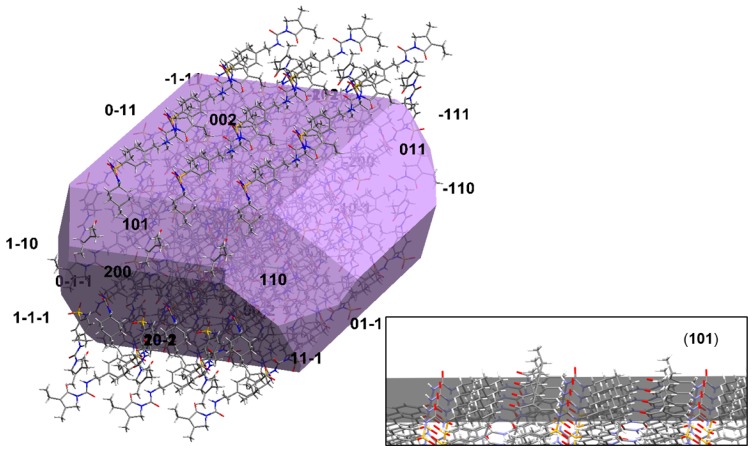
Crystal morphology of glimepiride calculated according to the attachment energy theory, featuring the orientation of a 3 × 3 supercell relevant to the crystal. The surface chemistry of the (101) Miller plane is illustrated in the insert.

Additional support of this view is provided by a thorough inspection of the crystal morphology of glimepiride, calculated according to the Attachment Energy (AE) theory, which is shown in Figure 8. A 3 × 3 supercell is superimposed on the crystal morphology model, in order to elucidate the surface chemistry of the morphologically most important faces.

According to the AE morphology model, initially the (101) plane constitutes only a small part of the overall crystal surface area, which is in agreement with the X-ray diffractogram of the raw (unmilled) material. The surface chemistry of the (101) slip plane, illustrated in Figure 8’s insert, shows that glimepiride exposes the hydrogen bond donor/acceptor atom pair responsible for the strongly hydrogen-bonded dimer formation. One would expect that this face, owing to the tendency to form dimers with dissolved molecules of glimepiride, would exhibit a high growth rate, due to the Ostwald ripening effect. It is reasonable to assume that the hydrogen bonding capacity of the (101) face is “saturated” by the nanosuspension stabilizers, which effectively block crystal growth and stabilize the nanocrystals. This seems to be particularly true for the two stabilizers that contain both hydrogen bond donor and acceptor groups (HPC and Pharmacoat^®^), and from those who lack hydrogen bond donors, only for poloxamer, whose ether oxygens are more accessible by the surface molecules of glimepiride, due to the absence of “bulky” substituents (such as rings) in poloxamer’s structure. For the other two polymers that lack hydrogen bond donor groups, namely PVP K25 and Soluplus^®^, the presence of bulky substituents and the complex branched structure seem to limit their ability to act as stabilizers, as has been evidenced by the increasing particle size of the nanocrystals during milling (Figure 2b).

Moreover, the formation of hydrogen bonds between glimepiride and stabilizers on the overexpressed (101) face could explain the observed milling-induced changes in the FTIR absorption bands discussed above.

#### 3.3.5. Scanning Electron Microscopy (SEM)

**Figure 9:**
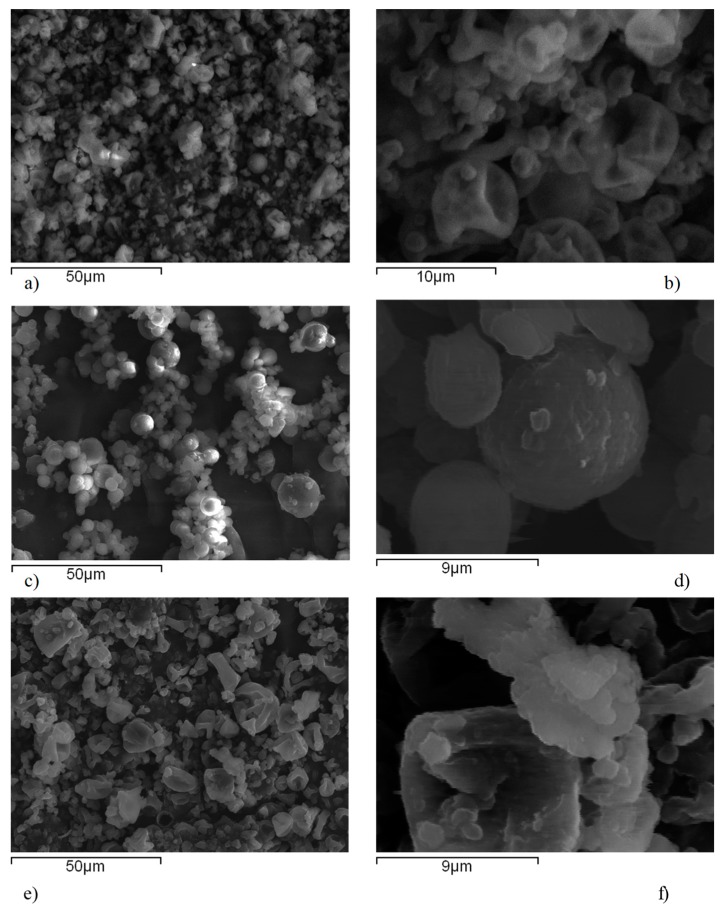
SEM photomicrographs of spray dried glimepiride nanosuspensions: (**a**,**b**) F1 (HPC-SL), (**c**,**d**) F2 (poloxamer 188), and (**e**,**f**) F3 (Pharmacoat^®^ 603).

Scanning electron micrographs of spray dried nanosuspensions are shown on Figure 9. Spray dried particles of nanosuspensions stabilized with HPC-SL and Pharmacoat^®^ 603 showed characteristic wrinkled-surface sphere morphology. During spray drying of droplets, spherical particles are initially forming a thin solid coat during drying of the droplet surface, under which the liquid droplet interior is still preserved. Progression of drying process causes elevation of pressure within the sphere due to water evaporation from interior droplet, which leads to the collapse of the sphere and formation of particles with hollow surfaces [53]. Spherical agglomerates were observed in the micrographs of spray dried nanosuspension stabilized with poloxamer 188, attributed to the partial melting of poloxamer in the stream of hot air during the spray drying process. This hinders redispersion of nanoparticles upon contact with aqueous medium, as proven by the results of redispersibility testing, probably due to a thick gel later formation. A close-up look at the particle surface (Figure 9b,d,f) revealed the presence of individual nanoparticles embedded within the mannitol matrix.

#### 3.3.6. In Vitro Dissolution Testing

**Figure 10:**
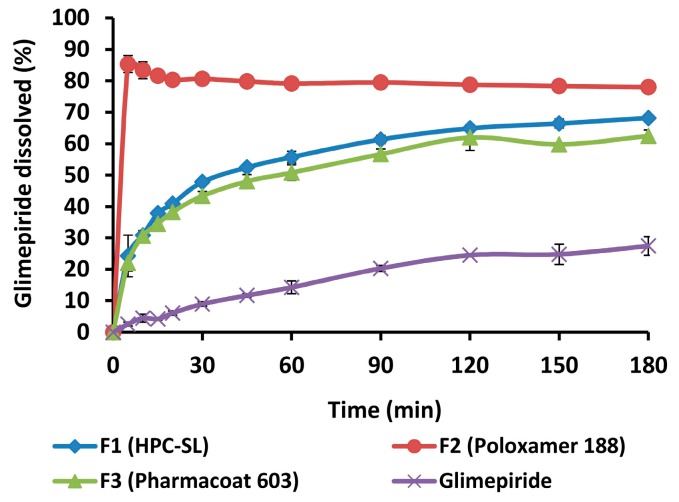
Dissolution profiles of glimepiride from the samples of pure drug and spray dried nanosuspensions.

Dissolution profiles of pure glimepiride and spray dried nanosuspensions are shown in Figure 10.

Pure glimepiride showed very slow and incomplete dissolution with less than 30% of dissolved amount during 3 h of testing. This behavior of pure glimepiride is expected as it is practically insoluble within the physiological pH range, with a reported solubility of 0.0012 mg/mL in aqueous media with pH 7.0, and 0.00087 mg/mL in pH 6.8 [24]. Glimepiride dissolution rate from all three formulations of spray dried nanosuspensions was improved compared to that of pure glimepiride. Dissolution testing clearly showed the superior performance of nanosuspension stabilized with poloxamer 188, although this sample showed the lowest redispersibility due to particle aggregation during spray drying process. This apparent discrepancy between dissolution rate and redispersibility result can be explained by the fact that poloxamer 188 exhibits typical surfactant properties, thus providing enhanced wetting of glimepiride particles. This makes the surface of glimepiride particles more hydrophilic facilitating interactions with the dissolution medium and further promotes dissolution of glimepiride particles. These results showed that in the case of spray dried glimepiride nanosuspensions, the intrinsic properties of the stabilizer rather than the size of redispersed particles determines dissolution of glimepiride.

## 4. Conclusions

Production of nanocrystals of glimepiride with a uniform particle size distribution is feasible by wet media milling, with the aid of poloxamer 188 and low viscosity grades of HPC and HPMC polymers. Wet media milling proved advantageous technique for production glimepiride nanosuspension in that it does not induce polymorphic transformations, preserving the stable polymorph of glimepiride. Spray drying is an efficient solidification method, which stabilizes the nanocrystals and ensures redispersibility when appropriate stabilizers are used. Lattice energy framework combined with topology analysis and crystal morphology modeling can provide significant insight into the mechanisms of particle fracture and provide explanations for otherwise difficult to understand experimental findings. Finally, dissolution testing proved the enhanced dissolution of nanocrystals, and additionally revealed the fact that redispersibility alone is not a good predictor of dissolution rate, but the stabilizers’ intrinsic properties should always be taken under consideration.

## Figures and Tables

**Table 1:** Particle size (z-average) and polydispersity index (PDI) of glimepiride suspension after 60 min of milling and after 7 days storage in a refrigerator (Mean ± SD).

Stabilizer	Particle Size (nm)	PDI	Particle Size (nm) after 7 Days	PDI after 7 Days
HPC SL^®^	207.7 ± 1.8	0.213 ± 0.007	183.3 ± 6.6	0.254 ± 0.025
Poloxamer 188	182.3 ± 11.8	0.181 ± 0.055	181.7 ± 3.5	0.196 ± 0.007
Pharmacoat^®^ 603	225.7 ± 11.7	0.209 ± 0.035	238.7 ± 5.7	0.248 ± 0.007
HPC L	326.7 ± 8.8	0.169 ± 0.020	311.3 ± 30.9	0.266 ± 0.039
Pharmacoat^®^ 615	397.7 ± 8.3	0.206 ± 0.011	379.5 ± 9.1	0.165 ± 0.015
PVP K25	1108 ± 156.4	0.597 ± 0.096	1715 ± 82.6	0.262 ± 0.069
Soluplus^®^	2008.3 ± 110	0.324 ± 0.090	1340 ± 23.4	0.265 ± 0.074

**Table 2:** Numbering of dimers, symmetry operation of second molecule of dimer and corresponding intermolecular interaction energy of dimers formed by the basic molecule in the crystals (dimers showing the strongest interaction are highlighted in bold).

Dimer	Symmetry Operator	Interaction Energy (kcal/mol)
1	1 + x, y, z 0 0	−8.7
2	1 + x, −1 + y, z 0 0	−3.1
3	−1 + x, y, z 0 0	−8.7
4	−1 + x, 1 + y, z 0 0	−3.1
5	1/2 − x, 1/2 + y, 1/2 − z 0 0	−12.0
6	1/2 − x, −1/2 + y, 1/2 − z 0 0	−12.0
7	3/2 − x, 1/2 + y, 1/2 − z 0 0	−2.2
8	3/2 − x, −1/2 + y, 1/2 − z 0 0	−2.2
9	−1/2 − x, 1/2 + y, 1/2 − z 0 0	−1.4
10	−1/2 − x, −1/2 + y, 1/2 − z 0 0	−1.4
**11**	**−x, 1 − y, −z 0 0**	**−17.5**
**12**	**1 − x, −y, −z 0 0**	**−21.5**
13	1 − x, 1 − y, −z 0 0	−11.1
14	2−x, −y, −z 0 0	−2.7
15	1/2 + x, 1/2 − y, −1/2 + z 0 0	−6.2
16	−1/2 + x, 1/2 − y, 1/2 + z 0 0	−6.2
